# “Who blends in and why (not)?” A qualitative study on psychotherapists' patient inclusion in blended care

**DOI:** 10.1016/j.invent.2025.100847

**Published:** 2025-06-18

**Authors:** Sophie Jordan, Pauline Becker, Solveig Behr, Friederike Fenski, Christine Knaevelsrud, Johanna Boettcher, Carmen Schaeuffele

**Affiliations:** a Freie Universität Berlin, Dept. of Education and Psychology, Germany; b Psychologische Hochschule Berlin, Dept. of Clinical Psychology, Germany

**Keywords:** Blended care, Blended therapy, Qualitative content analysis, Internet-based intervention

## Abstract

**Introduction:**

Psychotherapists may act as bottlenecks in the integration of digital interventions into psychotherapy, known as blended care (BC). In the literature, various factors are discussed as potential inclusion, exclusion, or limiting criteria in BC.

**Method:**

Our aim for this interview study was to gain a deeper understanding of the factors psychotherapists consider when inviting patients to participate in BC. For this purpose, we interviewed seven psychotherapists with a psychodynamic and seven psychotherapists with a cognitive behavioral background who participated in a naturalistic trial on BC in routine outpatient psychotherapy.

**Results:**

Psychotherapists considered few fixed inclusion or exclusion criteria when considering which patients to introduce BC to. The basic technical requirements had to be met and the patients had to be “fit for outpatient therapy”. Psychotherapists found patients' response to BC, like their motivation, to be a decisive factor when considering BC.

**Discussion:**

Psychotherapists emphasized patient motivation for BC as a potential bottleneck in its implementation. Therefore, a successful implementation strategy should focus on strengthening both psychotherapists' and patients' motivation to engage with BC. The openness of psychotherapists towards patient characteristics suggests that BC in outpatient care may target a broad patient population.

## Highlights

• Few differences were found between psychodynamic and CBT psychotherapists in their decision making in introducing blended care
• Psychotherapists apply very few hard inclusion or exclusion criteria when introducing blended care
• Patient motivation, a safe work environment and internet literacy were seen as prerequisites by psychotherapists for introducing blended clare
• Patient who are fit for outpatient therapy were generally seen as being fit for blended care by psychotherapists
• Psychotherapists noted that establishing a stable therapeutic relationship could facilitate the introduction of blended care

## 1 Introduction

The growing adoption of digital interventions is altering the psychotherapeutic landscape. While there is substantial evidence supporting digital interventions' efficacy as standalone treatments (Taylor et al., 2021), the integration of digital tools with traditional face-to-face psychotherapy — known as blended care (BC) — remains less explored. First studies indicate that BC is an effective treatment, particularly for depression (Berger et al., 2018; Ferrao Nunes-Zlotkowski et al., 2024; Lungu et al., 2020; Owusu et al., 2022; Romijn et al., 2021). BC may have several advantages: BC enables around the clock access to digital therapeutic content, to potentially deepen the process outside of f2f sessions (Titzler et al., 2018; Urech et al., 2019). Digital interventions can also provide a structure that allows patients to work on content themselves, which can help to improve patients' self-management skills and empowerment (Cerga-Pashoja et al., 2020; Richards et al., 2018; Sander et al., 2022). By delivering content such as psychoeducation through digital interventions, BC can free up valuable time in face-to-face sessions to focus on acute topics and more personalized care (Bielinski et al., 2022; Phillips et al., 2021; Toonders et al., 2021). As a result, BC has the potential to both intensify therapy and offer more efficient and scalable mental health care solutions.

Psychotherapists in Germany seem cautiously interested in BC, as only a few psychotherapists have actual experience with BC (Phillips et al., 2021; Sander et al., 2022). Germany has only recently established a legal framework for digital health applications (Gerlinger et al., 2021). Compared to countries like the UK and the Netherlands, it lags behind in terms of integrating digital interventions into its healthcare system. Accordingly, training and experience in applying BC in psychotherapists is still limited (Gerlinger et al., 2021; Mendes-Santos et al., 2022; Titzler et al., 2018).

Who blends in? – Therapists' Considerations of which Patients to introduce BC to Taking a closer look at psychotherapists' reservations regarding BC is important, as they serve as gatekeepers regarding the introduction and implementation of BC (Schröder et al., 2017; Schuster et al., 2018). Psychotherapists rely on their prior experience with digital interventions and BC when deciding which of their patients to offer BC to: Studies show that less experienced psychotherapists tend to express greater skepticism about the feasibility and relevance of BC for their patients than more experienced psychotherapists (Mol et al., 2019; Schuster et al., 2020). Several factors, such as severe symptoms, psychotic experiences, personality disorders, trauma, comorbidities, and suicidal ideation, are seen as potential barriers to a patient's suitability for BC by therapists (Kip et al., 2020; Mendes-Santos et al., 2022; Mol et al., 2019; Titzler et al., 2018; Wentzel et al., 2016). Additional patient characteristics may limit suitability for BC according to psychotherapists include low cognitive abilities, insufficient computer skills or access to technology, and an insecure home environment (Kip et al., 2020; Mol et al., 2019; Sander et al., 2022; Wentzel et al., 2016). Patients' motivation was another factor that has been highlighted in numerous studies (e.g. Sander et al., 2022; Toonders et al., 2021). Conversely, patients considered suitable for BC are typically seen as younger and presenting with less severe symptoms (Mendes-Santos et al., 2022; Mol et al., 2019; Sander et al., 2022). Experienced psychotherapists, however, believed that the criteria for eligibility for BC and the therapeutic relationship in BC did not differ substantially from f2f therapy (Mol et al., 2019). Therapists highlight the importance of their autonomy in deciding whom to offer BC to and the need for shared decision-making (Kip et al., 2020; Titzler et al., 2018). For this purpose, the “Fit for Blended Care” instrument was developed (Kip et al., 2020; Wentzel et al., 2016). It aims to support decision-making for therapists when engaging patients in BC interventions and collates many of the aspects discussed above, whether anticipated or experienced. The instrument was developed through focus groups and in-depth interviews with patients and psychotherapists (Wentzel et al., 2016) and refined in a later study for forensic psychiatric outpatients (Kip et al., 2020). Using this instrument, basic practical requirements, as well as patient-related factors such as motivation and reflectiveness, can be discussed with the patient. In outpatient care reality, however, the first question is whether therapists would even consider BC for their patients, and to which patients they would offer BC in the first place.

### 1.1 The current study

This study aimed to achieve a deeper understanding of CBT and psychodynamic psychotherapists' decision-making processes in patient selection for BC in routine outpatient psychotherapy through a qualitative approach. For this purpose, a subsample of psychotherapists within a randomized controlled trial on BC in routine care was interviewed (Schaeuffele et al., 2022). Psychotherapists could decide which of their patients they invited to participate in this naturalistic study on a transdiagnostic, modular and transtheoretic BC intervention in routine psychotherapy. Thus, psychotherapists had the choice to offer BC to a patient or not, reflecting real-world practice.

## 2 Method

### 2.1 Trial design and sample

This interview study is part of a randomized controlled trial on the effectiveness of BC with online transdiagnostic modules in routine psychotherapy (German clinical trials registration: DRKS00028536). The effects of BC in routine care were compared to routine psychotherapy, i.e. treatment as usual (TAU), in Germany. More information is provided in the study protocol of the main study (Schaeuffele et al., 2022). The ethics committee of Psychologische Hochschule Berlin (EK202121) approved the interview study. All participants provided informed consent.

### 2.2 Intervention

“TONI” is a modular, transdiagnostic digital intervention that incorporates methods of different psychotherapeutic approaches. The intervention was developed in a participatory approach, including psychotherapists' and patients' perspectives in the design process (Behr et al., 2024). The result is a digital tool of 12 different modules targeting different areas of treatment, in detail described in the study protocol (Schaeuffele et al., 2022).

When recruiting psychotherapists to participate in the study, TONI was depicted as a flexible, modular online toolbox that can be flexibly adapted to fit adult patients with diverse psychological concerns across the whole spectrum of mental health. When psychotherapists signed up for the study, they received information materials via mail, including a manual outlining the content of the TONI modules and tips on how to integrate TONI into psychotherapy. Psychotherapists were also provided with prerecorded introductory videos when first signing up to the study. In addition, live online training sessions were offered via videoconferencing to help psychotherapists to get to know the platform and answer any questions they might have.

Patients could participate in the study when they met the following criteria: (a) over 18 years old, (b) internet access, (c) sufficient knowledge of German and (d) able to read and write. Psychotherapists were free to decide which of the modules to use with which patient. They “prescribed” the digital content. Psychotherapists were also free to decide whom to invite to the study, thereby making a choice on which patients they deem eligible for BC. However, they had to decide in the very first sessions, as the study design required early inclusion (or exclusion).

### 2.3 Participants

**Fig. 1:**
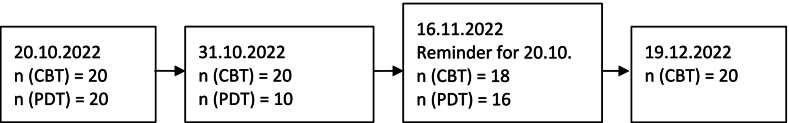
Recruiting process. *Note*. n refers to the number of psychotherapists contacted to participate in the study.

In the present qualitative study, we targeted a sample size of 6–10 psychotherapists for CBT and PDT each, following recommendations of data saturation in qualitative interview studies (Guest et al., 2020). Psychotherapists had to be registered in TONI at least four weeks before the interview to participate in the sub-study (*n* = 197). In two rounds, which took place 11 days apart, a total of 70 psychotherapists (n(PDT) = 30, n(CBT) = 40) were randomly selected from the population of 185 therapists who met the selection criteria. Those who had not yet responded in the first round were contacted again on November 16 and reminded of our request. In addition, a further 20 CBT therapists were drawn by lot on December 19, as we had received very little feedback from them. Psychotherapists were informed about the procedure and objective of the interviews. By agreeing to participate, they agreed to study conditions and gave informed consent. After seven interviews in both therapeutic approaches (CBT & PDT), data saturation was reached and we ended the recruitment process. The recruiting process is shown in Fig. 1.

### 2.4 Data acquisition

To obtain an understanding of the psychotherapists' decision-making processes in patient selection, we developed a semi-structured interview guide based on the BC literature, especially on the “fit for blended care” instrument (Kip et al., 2020; Wentzel et al., 2016). Commencing with open questions about the general selection process, the interview continues with a structured part based on previous research findings on patient selection for BC. As the perception of BC also depends on the background of the psychotherapists, their experiences with and motivation for study participation were taken into account. Besides, we investigated process-related factors such as the therapeutic alliance, temporal aspects, and features of the BC program. These aspects were discussed as barriers and facilitators for BC implementation in literature and therefore supposedly influence patient selection. For details see the interview guide in Appendix A.

The interview guide was tested with psychologists and modified three times from June to September of 2022. The interviews relevant to the study were conducted between October 2022 and January 2023. During the interview, the guideline questions were read out. Depending on the participants' previous answers some questions were adapted, for example by referring to what had already been said. At times participants were asked to clarify or to elaborate on their answers. The average duration of an interview was 31.02 min (SD = 5.58, range = 26.1 min; 41.52 min). The interviews were carried out via video call, where they were recorded and saved as audio files.

### 2.5 Data analysis

The interviews were transcribed, anonymized, and analyzed. The interview data was structured and analyzed following a deductive-inductive qualitative content analysis approach as described in Kuckartz (2016) using the software MAXQDA 2022 (VERBI Software, 2021). Therefore, a deductive category system was set up based on the structure of the interview guide. After familiarization with the interview data, both main authors independently coded the longest interview of each therapeutic approach and thereby generated first (sub)categories.

The main authors SJ and PB then discussed and adjusted the category system and repeated the process by coding two more interviews each and revising the category draft in consensual meetings. During the consensus discussions, a two-dimensional coding system was developed, including a valence category that could be applied in addition to each (sub)category. This labeled information about how the psychotherapists assessed the influence of the discussed variables on the selection process. Based on approximately 40 % of the interview material, we developed a codebook containing a definition, rules of application and anchor examples for every (sub-)category (for detail see Appendix B). The process was repeated iteratively, i.e. every time the codebook was adjusted, all interviews were recoded. Participants were not asked to provide feedback on the code system.

To ensure the codebooks' reliability, both main authors independently coded a randomly selected interview of each author and calculated the intercoder coefficient kappa allowing a tolerance range for segment overlap of 80 %. Following Kuckartz (2016) we calculated Cohen's kappa and achieved a good interrater-reliability (κ = 0.72–0.73). Nevertheless, we discussed the occurring discrepancies and refined the codebook again. Eventually, all interviews were coded using the revised category system. To provide a descriptive overview of shared topics among the interviewees, we also reported frequencies of codes.

## 3 Findings

### 3.1 Coding system

In a deductive approach, we derived three main categories (level 1) and eleven level 2 categories from the literature for factors that might influence therapists' decisions to offer BC treatment to patients. Main categories were: *patient-related factors; therapy and study conditions; therapist-related factors*. The coding system is shown in Appendix B. Two of these main categories, namely *patient-related factors* and *therapy and study conditions,* directly address patient selection and are the focus of the analysis. *Patient-related factors* cover all characteristics of the patients that influence the selection process of psychotherapists including *demographic variables*, *living conditions* as well as *symptoms,* and *patients' preconditions* (basic requirements to be able to use BC). *Therapy and study conditions* include the organizational framework of the therapy and study, as well as content-related and therapeutic aspects of the TONI platform and the *therapeutic alliance*. The third section, namely *therapist-related factors,* provides insight on the samples' experience with and attitudes towards BC. Thirty-seven inductive subcategories (level 3) emerged during the coding process. We will report frequencies of subcategories (level 3) for CBT and PDT psychotherapists separately to shed light on potential differences.

#### 3.1.1 Valence dimension

To represent patient selection more precisely, a second dimension was added to the coding system. The *valence* dimension was assigned additionally to the codes which focus on patient selection, namely *patient-related factors* and *therapy and study conditions,* and consists of five categories. A *prerequisite* indicates that a certain factor was required for successful implementation of BC. Likewise, *(rather) exclusion* was assigned when a *prerequisite* is not given or when the presence of a factor makes it considerably more difficult to use BC, so the psychotherapist would not include patients in BC. *Usage adaptation*, in turn, was assigned when a factor is considered a barrier but the psychotherapist can envision adjusting the use of BC accordingly, such as through closer supervision. *Indifferent* means that a characteristic was discussed without a clear direction of inclusion or exclusion or it was considered unimportant for the selection process. Finally, *particularly suitable* indicates that a circumstance or factor facilitates the use of BC or that BC may be particularly indicated in these cases.

### 3.2 Participant characteristics

In the present study, 14 psychotherapists, eight of them women, agreed to participate in the interview study. Seven of them were licensed in CBT and seven in PDT, including four child and adolescent psychotherapists and two psychotherapists with additional systemic therapy qualifications. Psychotherapists were asked how many patients they had already invited to take part in the BC program. The range of included patients was none to eleven with an average of 3.2 patients. 10 of the 14 had already included at least one patient in the study. In general, the participating psychotherapists reported great openness when including patients for BC. Eight psychotherapists stated that they did not select patients at all but suggested BC to all patients who commenced psychotherapy with them (8/14). Despite the open invitation process, psychotherapists mentioned several factors that could potentially influence the invitation of patients to BC.

#### 3.2.1 Motivation to participate in blended care study

All psychotherapists (14/14) shared an *interest in expanding therapeutic possibilities*, which refers to a general openness in developing therapeutic options through BC. For instance, the psychotherapists were motivated by the fact that BC provided them with different methods or allowed increased flexibility in the design of the therapeutic process time- and content-wise. Besides, six psychotherapists commented positively on the intervention TONI. In addition, psychotherapists were motivated by *supporting research* in the field of BC (6/14).

#### 3.2.2 Previous experience with BC

The majority of psychotherapists (9/14) had *little experience* with BC prior to participating in the TONI study, three psychotherapists (3/14) had never used elements of BC in therapy. Two psychotherapists (2/14) routinely integrated BC in their therapy. By elements of BC, we refer to digital health applications, such as mindful apps or more comprehensive programs of mindfulness. Video therapy was not categorized as an element of BC. Current use of BC ranged from recommending a meditation app to using some digital elements in three-quarters of their psychotherapies. Psychotherapists provided mixed evaluations of their past experiences with BC. Seven therapists (7/14) reported BC had been *useful* in their therapeutic work and eight (8/14) reported *low participation* on the part of the patients.

### 3.3 Patient selection

In general, the participating psychotherapists reported great openness when including patients for BC. Some stated that they did not select patients at all but suggested BC to all patients who commenced psychotherapy with them (8/14). Regarding patient inclusion in the study, most therapists (10/14) had already registered patients for the study at the time of the interview, meaning that patients had actively accepted the invitation to participate in the study. In this way, self-selection of patients occurred, with patients actively declining the invitation to BC, incomplete enrollment, or later not using the program. Despite the open invitation process, therapists mentioned several factors that could potentially influence the invitation of patients to BC. In the following, we discuss the criteria mentioned by the majority of therapists of each therapeutic approach that play a role in the selection process (see Table 1). A detailed description of all code frequencies can be found in Appendix C.

**Table 1:** Frequently mentioned patient-related criteria concerning in- and exclusion to BC.

Code	n total (%)	n CBT	n PDT	Quotation
Patients' preconditions				
Fit for outpatient care	11/14 (79 %)	6/7 (86 %)	5/7 (71 %)	If someone is suitable for me to make use of psychotherapy, um, so if I take him into psychodynamic psychotherapy […] then he is also suitable for me to take part in it (T1).
Internet literacy	14/14 (100 %)	7/7 (100 %)	7/7 (100 %)	I could imagine where I wouldn't apply it would be with someone who I think has… he is not so technically versed (T3).
Participation motivation	12/14 (86 %)	6/7 (86 %)	6/7 (86 %)	If I have the impression that it could be exhausting to convince the person. Well, there was someone, a younger woman, who also had reservations about data protection and didn't want to find out more about it, and then I respected that and left it there, well, that contributed to it. (V5)

Symptoms				
Dissociative or psychotic symptoms (−)	9/14 (64 %)	4/7 (57 %)	5/7 (71 %)	If I were to work with someone with a psychotic disorder or very dissociative… Difficult. (T3)
Magnitude of acute and general distress (−)	10/14 (71 %)	6/7 (86 %)	4/7 (57 %)	But if there is such a big issue, where you have to use the first sessions to somehow provide support. Well, if that is the most urgent concern, first of all to create acute relief, so that they are able to take action again, that would somehow be a criterion for me to say, ok, then I wouldn't add that on top of it. And since it was in the study condition in such a way that one cannot offer it at a later time, they would simply drop out. (V6)
Severe trauma (−)	6/14 (43 %)	2/7 (29 %)	4/7 (57 %)	So if, for example, (…) (mh) (…) someone with post-traumatic stress disorder, where it is even more difficult to build up a relationship, (ehm) then I wouldn't want to put this additionally on them. And (ehm), yes, I would rather go without it (ehm) (V5).

Living conditions of patients				
Safe workstation	8/14 (57 %)	2/7 (29 %)	6/7 (86 %)	I‘d compare this to writing your diary. Working on these modules is something really personal and therefore, a safe space is needed.(T2)
Technical equipment	8/14 (57 %)	3/7 (43 %)	5/7 (71 %)	The only criterion for exclusion is, of course, if someone does not have the equipment with which they can do this. That has not been the case so far. (T1)
Time resources	10/14 (71 %)	4/7 (57 %)	6/7 (86 %)	Of course, in case of someone who I assume, I don't know, has small children or something like that, (ehm) that this could perhaps limit the frequency of the therapy, then I would probably consider even more quickly that video material could be helpful, yes, for self-study and so on, yes. But I haven't thought about it until now. But yes, so I would, or also other factors, limitations, physical limitations, (ehm) long distances or so, in which the frequency of therapy is limited (…). (V4)

Therapeutic alliance				
Stability of therapeutic alliance	13/14 (93 %)	7/7 (100 %)	6/7 (86 %)	So that we don't discuss the content at first, but first bring it back to the relationship level to see: “What has this done to us now?” And then decide whether we should look at it further or not. Because if it would disturb the relationship, then I wouldn't do it. (T3)

*Note*. Only codes that were mentioned by the majority of psychotherapists are presented (minimum 4 of 7 in at least one sample).

T 1–7 Participants of PDT sample.

V1–7 Participants of CBT sample.

(−) Criterion is seen as exclusion or as a barrier by the majority.

#### 3.3.1 Patient-related factors

##### 3.3.1.1 Patients' preconditions

Every psychotherapist (14/14) discussed *Internet literacy*, which refers to experience and confidence in using digital devices and platforms. For the most part, *Internet literacy* was taken as a given and thus *indifferent* or as a *prerequisite* and if not given as an *exclusion*. Another important patient-related factor was the patients' *participation motivation* for BC (12/14). Most psychotherapists (9/14) noted that patients' participation motivation was an essential prerequisite and a definite exclusion criterion if not given. They found that the intervention was only applicable if the patients themselves were motivated to use it. Besides, convincing or motivating the patient repeatedly would mean additional work for the psychotherapist. Moreover, the majority of psychotherapists rated the general *fit for outpatient care* among patients as a prerequisite for offering BC (11/14). This means that patients do not suffer from intense conditions, such as phases of mania, acute suicidality, or psychotic breaks, which would warrant more intensive care than outpatient psychotherapy.

##### 3.3.1.2 Symptoms

A recurring subject of discussion was the *magnitude of acute and general distress* (10/14). This refers to the level of stress influenced by psychopathology and life circumstances, as well as acute crises. The *magnitude of acute and general distress* also depends on the general resilience of the patient. Psychotherapists considered both high overall symptom distress as well as disorder-specific symptoms as possible exclusion criteria: 8/14 considered a high magnitude of distress as a reason for not offering BC because they feared that offering BC early in therapy may overwhelm patients and therefore preferred to introduce BC at a later stage in therapy. However, a small proportion of psychotherapists (3/14) also considered whether BC might be *particularly suitable* for patients with a high magnitude of distress, and thus a high level of treatment motivation, or to use BC as a form of intensive support. 9/14 psychotherapists considered *dissociative or psychotic symptom*s and 6/14 *severe trauma* as possible limiting factors.

##### 3.3.1.3 Living conditions

Patients' psychosocial conditions were mainly seen as a *prerequisite* for BC, e.g. in terms of *technical equipment* (8/14) and a *safe workstation* (8/14). An intrusive partner, no calm workplace and devices shared with others were considered as potential exclusion criteria. Likewise, *time resources* were considered a *prerequisite* leading to *exclusion* (6/14) or *usage adaptation* (2/14) if not given. Besides this, two interviewees (2/14) discussed it as *particularly suitable* for patients with busy schedules to use digital components to fill gaps between f2f therapy sessions.

##### 3.3.1.4 Demographics

Psychotherapists did not state any definitive inclusion or exclusion criteria for demographic characteristics. If they mentioned demographic factors, they described them as not considered for selection for BC. Psychotherapists mainly discussed *age* (11/14) but in relation to potential differences in Internet literacy*.* They considered the assumption that older people might lack Internet literacy or confidence, and that younger people are more familiar with the Internet and therefore better suited to it, to be a prejudice.

#### 3.3.2 Therapy and study conditions

##### 3.3.2.1 Role of intervention for psychotherapy

TONI is a transdiagnostic modular intervention that incorporates methods of different psychotherapeutic approaches. Therefore, it can be applied to a broad range of psychopathologies in outpatient care. With the code *intervention characteristics*, we refer to all potential factors of the intervention that psychotherapists might deem relevant for patient selection (e.g. offering BC to patients with sexual health issues as the tool offers a module on the subject). No particular content or feature was seen as critical to patient selection. The tool was perceived as fitting for all patients because content could be tailored to patients' needs (12/14). Psychotherapists appreciated *giving impulses* by doing BC (7/14). They described the intervention as opening the space for experimentation in a structured way outside f2f sessions - for psychotherapists and patients.

##### 3.3.2.2 Therapeutic alliance

The *stability of the therapeutic alliance* was discussed by most psychotherapists (13/14). As the interviewed study psychotherapists needed to decide within the first few sessions whether to offer BC to their patients or not, they discussed whether the therapeutic rapport was already sufficiently established to introduce BC. Respondents either stated that they had not yet established the alliance so early in treatment or that they would not admit patients to therapy if the therapeutic alliance was not stable enough. In addition, the *exclusion* of patients from BC was discussed when the digital element was perceived as a threat to the sustainability of the alliance (6/14). Examples of difficulties in developing the therapeutic alliance were acute crises, high mistrust, or personality disorders. Still, psychotherapists considered it possible to introduce BC at a later stage when a more stable relationship was built.

##### 3.3.2.3 Temporal aspects

Time constraints due to study conditions were seen as a barrier to the inclusion of patients in BC. The *moment of inclusion within the first few sessions* (a study inclusion criteria of the main trial) limited most psychotherapists (10/14) in their invitation process and thus influenced their selection. Apart from the restrictions of the study, most psychotherapists (8/14) evaluated BC as *particularly suitable* due to potential *time savings,* especially in case of limited time on the side of the patient or the psychotherapist. Increased time and flexibility were achieved by outsourcing content, bridging gaps until the start of therapy or in case of remote therapy. In addition, BC was seen as a way to intensify therapy in case patients wanted to progress quickly, had a high demand, or to open up the possibility to work on other contents in parallel to the f2f therapy.

### 3.4 CBT and PDT view on BC inclusion

While no conclusions can be drawn about systematic differences between CBT and PDT psychotherapists due to the qualitative approach and small sample size, we report selected code frequencies to illustrate the diversity of perspectives expressed in the interviews. Overall CBT and PDT psychotherapists described similar reasons for inviting or excluding patients to BC. Differences between the groups were mostly minor and qualitative in nature. For example, three PDT therapists had not previously invited any patients to use the platform, compared to one in the CBT group, although overall experience with BC was comparable across groups. Some potential divergence emerged in emphasis. For example, psychodynamic psychotherapists listed two symptom groups that were not or less frequently mentioned by CBT psychotherapists, namely *autism* (PDT: 2/7 vs. CBT: 0/7) and *severe trauma* (PDT: 4/7 vs. CBT: 2/7), although this may reflect the small sample size. Differences in used terminology also seemed to reflect orientation-specific frameworks:: PDT psychotherapists referred to *level of personality functioning* (3/7), while CBT psychotherapists emphasized the *ability to reflect* (2/7). Regarding the therapeutic alliance, PDT therapists seemed to more often expressed concerns about BC when the therapist–patient relationship felt fragile (PDT: 4/7 vs. CBT: 2/7).

Furthermore, PDT psychotherapists emphasized practical conditions such as a *safe work station* (PDT: 6/7 vs. CBT: 2/7) and *technical equipment* (PDT: 5/7 vs. CBT: 3/7). Regarding the reasons behind using BT: PDT psychotherapists emphasized BC's potential for *giving impulses* (PDT: 6/7 vs. CBT: 1/7), while CBT psychotherapists emphasized the potential of *time savings* (PDT: 3/7 vs. CBT: 5/7).

## 4 Discussion

This study aimed to provide insight into the decision-making process of psychotherapists when selecting new patients for BC. For this purpose, qualitative interviews were conducted with seven CBT psychotherapists and seven PDT psychotherapists from across Germany. The psychotherapists were participants in an RCT investigating a transdiagnostic BC intervention in routine care. The interviewed psychotherapists had limited prior experience with digital mental health interventions before the trial. They identified only a few clear inclusion or exclusion criteria for BC. Essential prerequisites for offering BC in an outpatient setting included patients' Internet literacy, suitability for outpatient care, and motivation to engage with the intervention. Other factors, such as specific symptoms and their severity, were not viewed as exclusion criteria but rather as elements requiring adaptation in the application of BC. The study provides a variety of perspectives on the implementation of digital mental health tools and helps to direct future steps.

A great openness towards BC in psychotherapists is an important and promising sign for future BC implementation (De Veirman et al., 2022; Drissi et al., 2023). The majority of our interviewees stated they did not select their patients for BC, but instead invited all patients commencing psychotherapy.

### 4.1 Basic practical requirements

The inclusion and exclusion criteria we found are mostly congruent with general facilitators and barriers for BC discussed in the literature (De Veirman et al., 2022; Sander et al., 2022; Schuster et al., 2020). In line with Wentzel et al. (2016) and Kip et al. (2020), most psychotherapists described basic requirements to use BC - specifically, technical equipment, a safe workstation, German language skills, and patients' time resources.

### 4.2 Motivation for BC

The interviewed psychotherapists particularly emphasized patient motivation. If there was no clear expression of the patient's willingness to participate in the study from the patients, BC was not implemented. Generally, Allan et al. (2022) found that patients must see the additional value brought upon by the intervention to be motivated to use it. On the part of psychotherapists, the motivation to use digital tools is enhanced by the perceived flexibility in terms of time and location as well as a positive expectation of results (Dockweiler et al., 2020; Phillips et al., 2021). In the current study, psychotherapists expressed positive expectancies such as increased flexibility and enrichment of the therapeutic process. However, they also reported that low patient motivation led to an increased workload as they additionally had to motivate patients, which might be an obstacle to the application of BC. Some practitioners stated that it was more difficult to motivate patients with severe and complex symptoms (Mol et al., 2019). This could lead to BC only being used with less severely impaired patients (Davies et al., 2020; Mendes-Santos et al., 2022), although a much larger group could benefit from it. Thus, expectancies were also influenced by the patients' motivation for BC and a potentially increased workload. This should be considered in intervention development to broaden the general implication of BC.

### 4.3 Fit for outpatient care

Psychotherapists discussed that patients needed to be fit for outpatient care. Indicators for exclusion of BC were in line with general criteria that psychotherapists consider when recommending other or additional services to outpatient psychotherapy. Factors limiting patient fitness included severe trauma, high levels of distress, and psychotic and dissociative symptoms. Expectations of alliance ruptures, dissociation, re-traumatisation, psychotic breaks and an inability to contain those contributed to psychotherapists being reluctant to include patients in BC. Previous studies summarized that “high symptom severity” was perceived as a barrier to BC (Kip et al., 2020; Mendes-Santos et al., 2022; Mol et al., 2019; Titzler et al., 2018; Wentzel et al., 2016). The results of our study suggest that the overall severity of symptoms might be less important than the disruptiveness of the symptoms. Therapists considered the use of BC to be difficult when patients might feel overwhelmed or if there was a risk that certain exercises could lead to an increase in symptoms.

In the main study, no inclusion or exclusion criteria were applied in regard to diagnoses or severity. It is striking that the exclusion criteria psychotherapists discussed relating to high-symptom distress or psychotic symptoms are consistent with typical study exclusion criteria in BC research. This shows that BC needs to be investigated for severe symptoms to possibly expand the target group. There are first indications from the perspective of practitioners (Bucci et al., 2019) as well as patients (Allan et al., 2022; Gumley et al., 2022) that BC interventions designed specifically for psychotic symptoms, e.g. hallucinations (Moore et al., 2020), can support psychotic patients and bring an added value to therapy. A scoping review by Van Der Boom et al. (2022) showed that BC for borderline personality disorder had no adverse effects and was accepted by patients. Disseminating such findings among psychotherapists is crucial so they can make evidence-informed decisions on inclusion.

### 4.4 Therapeutic alliance

Prior studies described the alliance as being similarly strong in BC and in classic f2f psychotherapy (Askjer and Mathiasen, 2021; Kooistra et al., 2019). Furthermore, psychotherapists' therapeutic alliance ratings were found to be predictive of the treatment success of BC (Vernmark et al., 2019). The therapeutic alliance was a crucial factor in the consideration of BC implementation, according to the psychotherapists we interviewed. Reflecting ongoing debates, they expressed a preference for first establishing a strong, sustainable therapeutic relationship before integrating BC into the process. They viewed this established alliance as essential for patients to truly benefit from BC. Some psychotherapists further reported constantly reflecting on the alliances' quality and considered interrupting or stopping BC if a threat to the alliance is perceived. Furthermore, in line with previous findings, it was pointed out that the digital tools can be an excellent bridge to reach some more withdrawn patients (Mol et al., 2019; Rasing et al., 2019) and can enhance positive real-life alliances between patients and psychotherapists (Moore et al., 2020).

### 4.5 Comparison of attitudes towards BC between PBT and CBT psychotherapists

First of all, it should be noted that in this study, CBT and PDT psychotherapists expressed similar concerns and benefits of BC in many aspects, including clear inclusion and exclusion criteria. Differences seemed to emerge primarily in the emphasis placed on certain factors. Practitioners of PDT were more focused on the impact of BC on the therapeutic process and its role in providing impulses. In both approaches, some psychotherapists viewed the digital intervention as a potential threat to the therapeutic relationship, particularly in patients with severe symptoms. However, PDT psychotherapists tended to explore these relational concerns in greater depth. For example, the possibility that patients may only engage in BC out of submissiveness or that BC may be particularly appropriate for withdrawn patients. CBT psychotherapists, on the other hand, focused more on organizational aspects, such as the possibility of working on content alongside regular sessions and possibly intensifying therapy. In addition, PDT psychotherapists discussed a greater variety of symptoms than CBT psychotherapists. Finally, the psychotherapists used different, albeit related, concepts: CBT psychotherapists partly considered the patients' ability to reflect, while PDT psychotherapists addressed the personality functioning of their patients.

### 4.6 Limitations

Several limitations need to be mentioned. First and foremost, our sample is not representative. We interviewed a small but diverse group of PDT and CBT psychotherapists. Self-selection influenced our recruitment firstly by only including psychotherapists who were already using BC. This group might have been inherently open to digital interventions and their use. They were also willing to participate in our interviews and expressed an interest in scientific research. Out of *N* = 100 psychotherapists who were invited to participate, only 14 % replied. In addition, the psychotherapists had limited experience with using BC due to the short practice period and the small number of patients they included in the RCT to date. Some statements were somewhat hypothetical and based less on experience. The justifications were often based more on the participants' gut feeling and less on objective criteria. Besides, the study reported frequencies for the specific codes. Due to qualitative approach and small sample, codes with a high frequency in the interviews cannot be understood as generally valid statements. They merely serve to provide a better descriptive overview of the interview data. Finally, the psychotherapists were very aware of the study guidelines. In particular, inclusion right at the beginning of psychotherapy was a theme in most interviews. The transfer to routine practice, therefore, sometimes seemed difficult. Future studies should investigate decision processes on offering patients BC outside a study context.

### 4.7 Conclusion

This interview study revealed which aspects psychotherapists reflect on when selecting patients for BC intervention. Psychotherapists are gatekeepers in the implementation of BC because, without their motivation, BC will not be applied. In the current study, psychotherapists were motivated for BC and open to invite all of their patients. The basic technical requirements had to be met and the patients had to be fit for outpatient therapy. Uncertainties about the use of BC arose with severely distressed patients. Nevertheless, offering BC was rarely seen as a threat to the therapeutic relationship. Mostly, psychotherapists found the patients' response to BC to be decisive. Consequently, patients' motivation for BC is another bottleneck in implementing BC. For a successful implementation strategy, strengthening psychotherapists' and patients' motivation to use BC are thus crucial target points. Future studies should investigate which factors influence usage motivation, particularly the motivation of patients. Psychotherapists' openness regarding patient fit suggests that BC may include a broad patient population, rather than preselecting younger or less severely affected individuals. Otherwise, prevailing assumptions about who benefits most from digital interventions—assumptions not yet well supported by empirical evidence—risk being unintentionally reinforced.

## Declaration of competing interest

The authors declare that they have no known competing financial interests or personal relationships that could have appeared to influence the work reported in this paper.

## Appendix A Supplementary data

Supplementary material Image 1
