# Supplementary material for: “Who blends in and why (not)?” A qualitative study on psychotherapists' patient inclusion in blended care
Source: Internet Interv. 2025 Jun 18;41:100847. doi: 10.1016/j.invent.2025.100847 (PMC12221746; doi:10.1016/j.invent.2025.100847)
Supplement: Supplementary file 1 — Supplementary material [file mmc1.docx]

**Appendix A**

**Interview guide for patient inclusion – English translation (original German version see below).**

**Introduction to the interview**

| Introduction |
| --- |

*"Thank you very much for taking the time for this interview!*

*The interview takes place in the context of my master thesis and I am interested in the decision-making process of which patients you invite to the TONI study. Before I get to the questions, I want to address a few organizational points:*

*For the analysis of the interview, we record this part of the conversation. The data will only be used for transcription and then be deleted. Do you consent to the recording?" -* Start recording

*"Since we are conducting these interviews with psychotherapists of different approaches, I may ask you for a brief explanation or clarification of mentioned psychotherapeutic concepts for my understanding.*

*The interview is expected to last 30 minutes, you may ask for a break at any time or end the interview without giving a reason.*

*Do you have any current questions?"*

| Motivation |
| --- |

*"In general, I am interested in knowing what motivated you to participate in the TONI study?"*

| Previous experience with blended care |
| --- |

*"Prior to the TONI study, have you ever used computer or mobile-based elements in therapy?"*

If yes:

*● "What applications did you use?"*

*● "How regularly did you use these applications?"*

*● "How would you rate this experience?"*

*"Do you currently have any patients enrolled in TONI?"*

*If yes:*

*● How many?*

| Patient-selection: decision process |
| --- |

*"Now we will talk in more detail about the selection of patients. You are inviting new patients to participate in the TONI study.*

*What goes through your mind when you consider whether to suggest a patient to participate in the TONI study?"*

1. **Focus: patient-related factors**

*● "Are there (any other) basic requirements concerning the patients that play a role for you in patient selection?"*

*● "Are there (any other) aspects of symptomatology that play a role for you in patient selection?"*

*● "Are there (any other) aspects of life situation that play a role for you in patient selection?"*

*● "Are there (any) other demographic aspects that play a role for you in patient selection?"*

After naming factors if not self-reasoned or elaborated:

*● "Why do these aspects play a role for you in patient selection?"*

*"Which of these would be aspects that would prevent you from suggesting to participate in TONI?"*

1. **Focus: therapeutic framework**

*"Up to this point we have rather looked at patient factors. The next step is to look at therapeutic frameworks."*

*● "To what extent does the therapeutic relationship play a role for you in patient selection?"*

*● "Are there (any other) time-related aspects that play a role for you in patient selection?"*

*(if asked: time spent before and after the session, integration in f2f therapy).*

*● "Are there (any other) contents or features of TONI that play a role for you in patient selection?" (modules, trackers, diaries)*

After naming factors if not self-reasoned or elaborated:

*● Why do these aspects play a role for you in patient selection?*

*"Which of these would be aspects that would prevent you from suggesting participation in TONI?"*

**Closing Decision Making**

*"Are there any other points you can think of about the patient selection decision-making process that you haven't mentioned yet?"*

If prior experience addressed:

*● "You said at the beginning that you have prior experience with computer- or mobile-based elements in therapy. To what extent does this experience play a role in your decision-making process?"*

| Actual inclusion and discrepancy |
| --- |

- - - 1. **If already patient included:**

*"We have now talked about different decision factors that might play a role in inviting patients. At the beginning, you said that you have already included patients. To what extent do you think there is a match between the patients you actually selected and the decision factors you just mentioned?"*

If there is a discrepancy:

*● "Where do you think this discrepancy might come from?"*

*"How do you think your use of TONI will evolve in the future?"*

- - - 1. **If no patients included yet:**

*"We have now talked about different decision factors that might play a role in inviting patients. At the beginning, you said that you haven't included anyone yet. How does that come about?"*

*"How do you think your use of TONI will evolve in the future?"*

| Conclusion |
| --- |

*"Thank you very much. From my side, that would be it, I have learned a lot.*

*Are there any more points you would like to share with me?"*

**Interviewleitfaden zum Patient:innen-Einschluss – deutsche Version (Original)**

***For English version see below***

| Einführung ins Interview |
| --- |

*“Vielen Dank, dass Sie sich die Zeit für dieses Gespräch nehmen!
Noch einmal kurz zum Ziel: Das Interview findet im Rahmen meiner Masterarbeit statt und mich interessiert der Entscheidungsprozess, welche Patient:innen Sie zur TONI-Studie einladen. Bevor ich zu den Fragen komme, noch ein paar organisatorische Punkte:*

*Für die Auswertung des Interviews zeichnen wir diesen Teil des Gesprächs auf. Die Daten werden lediglich zur Transkription verwendet und danach gelöscht. Willigen Sie in die Aufzeichnung ein?” -* Aufzeichnung starten

*“Da wir diese Interviews mit Psychotherapeut:innen verschiedener Verfahren durchführen, kann es vorkommen, dass ich Sie zu meinem Verständnis um eine kurze Erklärung bzw. Klarifikation erwähnter psychotherapeutischer Konzepte bitte.*

*Das Interview wird voraussichtlich 30 Minuten dauern, Sie können zu jedem Zeitpunkt eine Pause erbitten oder das Gespräch ohne die Angabe von Gründen beenden.*

*Haben Sie aktuell noch Fragen?”*

| Motivation |
| --- |

*“Ganz allgemein interessiert mich: Was hat Sie dazu bewogen, an der TONI-Studie teilzunehmen?”*

| Vorerfahrung mit Blended Care |
| --- |

*“Haben Sie vor der TONI-Studie schon einmal computer- oder mobilgestützte Elemente in der Therapie eingesetzt?”*

Falls ja:

- *“Welche Anwendungen haben sie genutzt?”*
- *“Wie regelmäßig haben Sie diese Anwendungen eingesetzt?”*
- *“Wie bewerten Sie diese Erfahrung?”*

*“Haben Sie aktuell bereits Patient:innen in TONI eingeschlossen?”*

Falls ja:

- *Wie viele?*

| Entscheidungsprozess der Patient:innenauswahl |
| --- |

*“Nun soll es genauer um die Auswahl der Patient:innen gehen. Sie laden im Rahmen der TONI-Studie neue Patient:innen zur Teilnahme ein.*

*Was geht Ihnen durch den Kopf, wenn Sie überlegen, ob Sie einem Patienten bzw. einer Patientin die Teilnahme an der TONI-Studie vorschlagen?”*

1. **Vertiefung: Patient:innenbezogene Variablen**
   - *“Gibt es (noch weitere) Grundvoraussetzungen der Patient:innen, die für Sie eine Rolle bei der Patient:innenauswahl spielen?”*
   - *“Gibt es (noch weitere) Aspekte der Symptomatik, die für Sie eine Rolle bei der Patient:innenauswahl spielen?”*
   - *“Gibt es (noch weitere) Aspekte der Lebenssituation, die für Sie eine Rolle bei der Patient:innenauswahl spielen?”*
   - *“Gibt es (noch weitere) demografische Aspekte, die für Sie eine Rolle bei der Patient:innenauswahl spielen?”*

Nach der Nennung von Faktoren falls nicht selbst begründet oder ausgeführt:

- *“Weshalb spielen diese Aspekte eine Rolle für Sie bei der Patient:innenauswahl?”*

*“Was davon wären Aspekte, die Sie davon abhalten, die Teilnahme an TONI vorzuschlagen?”*

1. **Vertiefung: Therapeutische Rahmenbedingungen​**

*“Bis hierhin haben wir uns eher Patient:innenfaktoren angeschaut. Im nächsten Schritt soll es um therapeutische Rahmenbedingungen gehen.”*

- *“Inwiefern spielt die therapeutischen Beziehung für Sie eine Rolle bei der Patient:innenauswahl?”*
- *“Gibt es (noch weitere) zeitliche Aspekte, die für Sie eine Rolle bei der Patient:innenauswahl spielen?”*(bei Nachfrage: Zeitaufwand vor und nach der Sitzung, Verzahnung in f2f-Therapie)
- *“Gibt es (noch weitere) Inhalte oder Funktionen von TONI, die für Sie eine Rolle bei der Patient:innenauswahl spielen?” (Module, Tracker, Tagebücher)*

Nach der Nennung von Faktoren falls nicht selbst begründet oder ausgeführt:

- *Weshalb spielen diese Aspekte eine Rolle für Sie bei der Patient:innenauswahl?*

*“Was davon wären Aspekte, die Sie davon abhalten, die Teilnahme an TONI vorzuschlagen?”*

**Abschluss “Entscheidungsfindung”**

*“Gibt es noch weitere Punkte, die Ihnen zum Entscheidungsprozess der Patient:innen-Auswahl einfallen, die Sie noch nicht genannt haben?”*

Falls Vorerfahrungen angesprochen:

- *“Sie haben anfangs gesagt, dass Sie schon Vorerfahrung mit computer- oder mobilgestützte Elementen in der Therapie haben. Inwiefern spielt diese Erfahrung eine Rolle in Ihrem Entscheidungsprozess?”*

| Tatsächlicher Einschluss und Diskrepanz |
| --- |

1. **Falls schon Patient:innen eingeschlossen:**

*“Wir haben nun über verschiedene Entscheidungsfaktoren gesprochen, die bei der Einladung der Patient:innen eine Rolle spielen könnten. Zu Beginn haben Sie gesagt, dass Sie bereits Patient:innen eingeschlossen haben. Inwieweit denken Sie, gibt es eine Übereinstimmung zwischen den von Ihnen tatsächlich ausgewählten Patienten und den eben genannten Entscheidungsfaktoren?”*

Falls es eine Diskrepanz gibt:

- *“Was denken Sie, woher diese Diskrepanz kommen könnte?”*

*“Wie denken Sie, wie wird sich Ihre Verwendung von TONI in Zukunft entwickeln?”*

1. **Falls noch keine Patient:innen eingeschlossen:**

*“Wir haben nun über verschiedene Entscheidungsfaktoren gesprochen, die bei der Einladung der Patient:innen eine Rolle spielen könnten. Zu Beginn haben Sie gesagt, dass Sie noch niemanden eingeschlossen haben. Wie kommt das dazu?”*

*“Wie denken Sie, wie wird sich Ihre Verwendung von TONI in Zukunft entwickeln?”*

| Abschluss |
| --- |

*“Vielen Dank! Von meiner Seite aus wäre es das, ich habe ganz viel erfahren.*

*Gibt es noch Punkte, die Sie mit mir teilen möchten?”*

# **APPENDIX B**

# **Coding System**

| **Code** | | **Definition** | **Application ("to code when"/"not to code when")** | **Anchor** |  |
| --- | --- | --- | --- | --- | --- |
| **Valence** | | Indicator whether the criterion is rather inclusion or exclusion criterion | - | *-* |  |
| prerequisite | | indicates that a criterion is necessary, thus a prerequisite for the use of BT | to code when therapists name characteristics that need to be given in the patient to offer them to participate | *So they have to be able to read, so they also have to be able to write without too much of a hurdle, and they also have to understand what is being presented (T5).* |  |
| (rather) exclusion | | indicates that a criterion is not suitable for the use of BT, BT would not be applied under this criterion | to code when therapist considers certain characteristics or circumstances to be necessary or unfavorable for blended therapy, for example, they would not introduce blended care under these circumstances | *So highly suspicious and somehow maybe even into... into paranoid symptoms. So I think I would think about it very carefully. No, so if I had the feeling that I would have to spend a lot of time explaining ne... how it works exactly, how data protection is and so on, I think I would... I probably wouldn't do that... do that. I think. (T2)* |  |
| indifferent | | Criterion was discussed, but not considered decisive for the selection | Main category to be coded indifferent when answer for question for main category is that it does not matter; Subcategory to be coded when the therapist has discussed a feature but concludes that the feature is not critical  not to code: if factor mentioned as "indifferent" but later turns out to cause usage adaptation / exclusion | *Exactly, it would just be the age, but there I would also, first of all, ask the people themselves, so, right? Because someone can be 80 and still be able to navigate well in the Internet, right? (V6)* |  |
| usage adaptation | | Criterion indicates a challenge for usage, and therapist is willing to adapt usage | to code when therapist considers certain characteristics or circumstances to be difficult for online therapy, but they say that the use could be adapted for it | *Of course, it depends on the situation: If someone is very suicidal or goes in that direction, you have to, I don't think that's impossible, but you have to take a close look at which modules you select and what might trigger too much or whatever. You can't control what happens in the time in between. But apart from that, I don't think there's anything to be said against it. So in the case of depressive symptoms, I would just take a careful look, especially at what I then select. (V7)* |  |
| particularly suitable | | Criterion indicates a particular eligibility or prerequisite for the usage of TONI platform, high chances therapists would invite patients with these characteristics to participate | to code when therapist considers certain characteristics or circumstances to be particularly suitable for blended care | *So basically everything that goes into the depressive, uh, corner, anxiety disorders... I would say that fits super well. (T7)* |  |
| **Therapist-related factors** | | Therapist's attitudes and experiences with IBI that might influence the selection process | **-** | **-** |  |
| **Previous Experience with digital Interventions** | | Use of IBI to date | - |  |  |
|  | none | never tried IBI before | to code when the therapist has never tried internet-based interventions before, even if the therapist mentions video therapy due to COVID-pandemia | *I: Before the TONI study, have you ever used computer- or mobile-based elements in psychotherapy? T4: No. No. (T4)* |  |
|  | little | already tried IBI in therapy a few times | to code when therapist states they have used internet-based interventions a few times, such as prescribing digital interventions (*DiGAs* are digital health applications that can be prescribed by health professionals in Germany) or working with meditation applications | *(Ehm) No, just a mindfulness app, the patients sometimes have mood tracker apps or something like that, which I integrate it a little bit, but actually not in a regular or structured way. (V2)* |  |
|  | routinely | IBIs an integral component of the therapy | to code when therapist states that internet-based interventions have already become an integral part of their therapy on a regular basis | *So um I regularly prescribed digital health apps and um exactly. Yes. But otherwise actually online surveys, online tests, such ... so more... more diagnostic tools (T5)* |  |
| **Evaluation of earlier Experiences** | | Evaluation of experience to date |  |  |  |
|  | low participation | Low participation on the part of the patients | to code when the therapist's previous experience was that internet-based interventions have not been well accepted by their patients yet, e.g. few patients participated or few have actively used it | *So, for example, I have prescribed DiGAs (ehm) a few times now and I also had the (..), I was surprised how few people actually used it. (...) I am also in a learning process. And (ehm), yes, it really surprised me, because I was also so really enthusiastic, and I can imagine, if it had affected me, that I would have done it immediately somehow and would have immediately tried somehow to get through it. But (ehm), it was less than I would have expected from the patients actually. (V4)* |  |
|  | useful | positive experiences and thus open attitude for further integration of digital intervention in therapeutic practice | to code when the therapist has previously found blended care useful in therapy in the sense that patients have benefited from it or it has opened up new possibilities for him/her | *V3: Well, it was (laughs) just the one case, that is of course a thin basis, data basis. (Ehm) Quite subjectively, I was amazed that the patient had benefited so much from it. I had not expected that, but she spoke very positively about it and was actually very taken with the, with this DiGa, yes exactly. (V3)* |  |
| **Motivation to participate/use TONI** | | therapist's motivation to participate in TONI study |  |  |  |
|  | positive attitude towards TONI | expression of positive associations with and expectations on TONI platform and/or study | to code when therapists show positive impression and expectations towards TONI due to e.g. development-process of the platform, the referral from BPTK, and other platform and study characteristics (transdiagnostic, modular, etc.) | *And then TONI came, and I thought "everything so free, everything so possible. I thought, great, great addition. That's what I was looking for.". And that definitely already played a role. (V1)* |  |
|  | supporting research | motivation to support research in the field of blended therapy | to code when the motivation for the study is supporting research in the field of blended therapy e.g. due to interest in evidence-based digital interventions | *So, on the one hand, I was moved by the fact that PDT is also being researched, I generally find that there is too little research on PDT (T2).* |  |
|  | interest in expanding therapeutic possibilities | general openness, interest in expanding therapeutic options through BC e.g. different methods, content, increased flexibility in the design of therapeutic process time- and content-wise | to code when the motivation for the study is that BC gives them new methods or media to work with, or BC enables a new way of influencing the patient, also to code when the therapist is motivated by increased flexibility through BC that enables e.g. remote work, bridging waiting time, longer intervals between sessions on side of the therapist | *And then this situation happened with Psycho-online and with the DiGAs, where I thought, yes, why not? Perhaps I can broaden my offer, just as I am learning another technique, I also want to broaden my offer. (V1)* |  |
| **Patients included** | | |  |  |  |
|  | | included | (some) patients have already registered in TONI platform | to code when (some) patients have already registered in TONI platform | *So, four have joined in now. I probably suggested it to eight. (V5)* |
|  | | not included | no patient has registered in TONI platform yet | to code when no patient has registered in TONI platform yet | *Well, I invited a patient a while ago, and I asked her again because she hadn't replied to the invitation yet. I checked again today and she still hasn't answered. (T6)* |
| **Patient-related factors** | | Characteristics of the patients influencing the selection |  |  |  |
| **Demographics** | | Characterizations of patients in the population and its structure based on objective factors | - | *-* |  |
|  | gender | the gender with which the patient self-identifies | to code when gender is mentioned as a factor of the selection process  not to code when examples of patients participating or stereotypes e.g. "an 80-year-old woman" are mentioned | *Not: (Ehm), I would not include, now so as a cliché image, the old lady, 80 years, who can see badly, who (ehm) has memory problems and where the man always sits there and constantly asks, "What are you doing now?". "Why are you on the computer now", or on the laptop, yes. That would now be like that, would now be a clichéd summary. That is already sufficient now as a summary, actually. (V3)* |  |
|  | age | numeric and/or developmental age of patients | to code when the patient's age is mentioned as a factor of the selection process, including mentioning the study inclusion criterion (min. 18 years) not to code: lack of certain abilities influences or is associated by the patient‘s age | *It depends on their maturity. So I would decide based on maturity. If it was open for adolescents, there are 14-year-olds for whom I could envision some of the content. (T7)* |  |
| **Living Conditions of Patients** | | psychosocial conditions influencing the selection | - | *-* |  |
|  | technical equipment | availability of computer or smartphone and internet access to work on digital content. | to code when technical equipment like a smartphone, including mentioning patient‘s (limited) financial resources leading to (in)availability of devices or internet access  not to code when the therapist mentions that devices are shared (then: safe workstation) | *The only criterion for exclusion is, of course, if someone does not have the equipment with which they can do this. That has not been the case so far. (T1)* |  |
|  | safe workstation | Place where the content of the platform can be worked on in peace and in privacy | to code when necessities of the situation in which the patient can work on TONI are mentioned, such as privacy and tranquillity, also the necessity for the patient to have their own device when it comes to being able to work on BC in tranquillity and privacy  not to code when technical equipment and internet access are described without mentioning aspects of privacy or safe environment | *I‘d compare this to writing your diary. Working on these modules is something really personal and therefore, a safe space is needed. (T2)* |  |
|  | time resources | time resources on the part of patients | to code when time resources or limits on side of the patient are mentioned referring to having time for attending f2f therapy or to work on TONI content, including discussion of distance to therapist's office  not to code when time resources and constraints of the therapist are mentioned, not to code when time refers to travel distance to therapy, not to code when time refers to the interval between sessions on side of the therapist | *Of course, in case of someone who I assume, I don't know, has small children or something like that, (ehm) that this could perhaps limit the frequency of the therapy, then I would probably consider even more quickly that video material could be helpful, yes, for self-study and so on, yes. But I haven't thought about it until now. But yes, so I would, or also other factors, limitations, physical limitations, (ehm) long distances or so, in which the frequency of therapy is limited (...). (V4)* |  |
| **Symptoms** | | Diagnoses or symptoms of the patients influencing the selection | - | *-* |  |
|  | anxiety and depression | Symptom complex including criterion of depression and/or of anxiety diagnosis | To code when mentioned that patient suffers mainly from depression or anxiety disorder | *On the other hand, also the problems that the patients bring. Those are often related to depressive attitudes, adjustment disorders, conflicts with self-worth, those things. And then I think that's where TONI offers good material. (V1)* |  |
|  | autism | patients that are diagnosed with Autism Spectrum disorders | to code when therapists mention the diagnosis of ASD as criterion for inclusion or exclusion due to possible effects the intervention might have on the patient | *I could imagine autism might be... I‘d rather not apply it with these patients because… yeah, I‘d rather wait for study findings there. (T7).* |  |
|  | dissociative or psychotic symptoms | Possibility that patient may dissociate and or shows psychotic symptoms such as hallucinations, delusions or confused and disturbed thoughts | To code when mentioned that patient is at risk to dissociate during the course of treatment and when therapist mentions psychotic symptoms such as hallucinations, delusions or confused and disturbed thoughts | *It would certainly be different if I treated (ehm) psychotic patients on an outpatient basis. Then you could think about it again, if they are in such an acute phase, whether I would offer it to them. (V6)* |  |
|  | magnitude of acute and general distress | level of stress resulting from general resilience, which is influenced by psychopathology and life circumstances, as well as from acute distress due to crisis | to code when the patient's level of distress is mentioned, which depends on the patient's ability to cope with distress. This refers to both acute crisis and chronic distress (e.g. high symptom load, death of a close person, conflicts,..)  not to code: when symptomatic explanation for stress is given that can be coded with other category of symptoms (psychotic/ dissociative symptoms, severe trauma, anxiety and depression, media addiction) | *But in such existential crises... I think it is somehow also important that there is this human encounter and that one is first always very close to what is needed now. (T2)* |  |
|  | media addiction | significantly increased media consumption, including high amounts of Screentime hours / per week | to code when media dependence is suspected in the patient | *So when I have young people with a significantly increased media consumption... So we are talking about those who are not in school, or who attend school or training irregularly, we are talking about over 100 hours per week in some cases. That's where I say I don't want to encourage them by pointing them to an app so that they can legitimise their high media consumption. I would rather keep my distance if I thought it had the character of addiction. (T6)* |  |
|  | level of personality functioning | concept found in Alternative SDM-5 Model of personality disorders (AMDP), including four core personality elements using a self and interpersonal framework comprise the dimensions, describing personality structure | to code when therapists mention organizational level and resulting psychological functioning, such as self-regulation, interactional patterns etc. | *Anyway, in retrospect, there are two patients to whom I would have said, "I wouldn't have offered it to them". Because they had such a borderline structure where I could imagine that they might have felt devalued if I had suggested something like that, where it... where they might have had the feeling "she doesn't want to see me" or something. (T3)* |  |
|  | severe trauma | traumatic experiences in the patient's biography and why these can influence the selection process | to code when therapist suspects severe trauma or post-traumatic stress disorder | *So if, for example, (...) (mh) (...) someone with post-traumatic stress disorder, where it is even more difficult to build up a relationship, (ehm) then I wouldn't want to put this additionally on them. And (ehm), yes, I would rather go without it (ehm) (V5).* |  |
| **Patients' preconditions** | | Non-pathologic patient variables | - |  |  |
|  | ability to reflect | Ability of metacognitive processes their thoughts, feelings and behavior, "meta-cognitive process" of reflecting on thoughts, emotion and behavior | to code when therapist discusses the patient's ability to observe and work on their thoughts, feelings and behavior, "meta-cognitive process" of reflecting on thoughts, emotion and behavior   not: cognitive abilities or dissociative and / or psychotic | *And if I have a little more space, if I have understood the patient's problem relatively quickly, if I also have the feeling, it is actually something (ehm) where cognitive work can be applied well, yes. Maybe also this thought, how much influence do I have on my life, how much influence do I have on my thoughts, yes, and how can I influence something. If it doesn't seem so completely unfamiliar to someone, then it's easier for me to dock on. But if that is still totally strange, then I would have, I think, a lot of fear that I hurt people by, by not making them feel taken seriously. Well, because, it would be a misunderstanding, but that the misunderstanding could come up. (V4)* |  |
|  | cognitive abilities | Patient's ability to process, reason, and to solve problems, so understanding contents and exercises | capability of patients to understand TONI tasks and contents and ability to work on those | *Having limitations in the ability to write and intellectual deficits... I don't know, an IQ of under 85. Where I would guess that you, I have read through this before, they might be overwhelmed by it (T5)* |  |
|  | fit for outpatient care | Whether outpatient care is indicated | to code when therapist discusses whether patient is suitable for outpatient therapy rather than an inpatient setting or other form of care  not: ability to reflect; fluent in German written and spoken | *I think I would not do it now with someone with a psychotic disorder (ehm) I would not use it (ehm). (5 sec) Yes, only then, so you have to check to what extent psychotherapy is possible. And I would be very cautious, if there are suicidal tendencies. (ehm) yes. (V5)* |  |
|  | fluency in German (written and spoken) | Patient is able to speak, read and write in German | to code when therapist mentions the ability to speak, read and write in German | *So basically, of course, it will be necessary for someone to be able to read and write, and to have sufficient mastery of the German language, so the language is a basic prerequisite (T4)* |  |
|  | Internet literacy | Experience and confidence in using digital devices and digital platforms | to code when therapist talks about the patient's ability to navigate through the internet, for example: patient feels comfortable or insecure in using online applications | *I could imagine where I wouldn't apply it would be with someone who I think has... he is not so technically versed (T3)* |  |
|  | participation motivation | Willingness and interest of the patients to use TONI platform and to participate in the study | to code when therapist mentions patient's attitude toward using digital interventions: open, motivated, or does not want to participate e.g. due to doubts about data security, lack of interest | *If I have the impression that it could be exhausting to convince the person. Well, there was someone, a younger woman, who also had reservations about data protection and didn't want to find out more about it, and then I respected that and left it there, well, that contributed to it. (V5)* |  |
|  | therapeutic experience | The patient's knowledge of therapeutic procedures and psychoeducational content of previous therapies | to code when therapist mentions the patient's therapy experience, i.e. the extent to which the patient is familiar with the framework, content such as psychoeducation or therapeutic exercises   not: lack of time (Time resources), insecurity in internet usage (internet literacy), high distress (symptoms - > distress) | *If someone comes to their third psychotherapy and actually already knows quite a lot and it is more about implementing things, then I don't think I would start offering them all kinds of information again. If someone is in therapy for the first time - as is the case with the current candidate - then I can imagine it better (T4)* |  |
| **Therapy and Study Conditions** | | General conditions of the study design, platform and temporal organization of the therapy | - | *-* |  |
| **Role of Intervention for Psychotherapy** | | Added value of TONI for therapy. This refers to both the content and the effects of using TONI platform in therapy. (Self-efficacy, new points of view, transfer to everyday life, outsourcing or intensification of psychoeducation). | - | *-* |  |
|  | give impulses | Provide other points of view to patients in their everyday life, possibility to have influence outside of therapy in everyday life as well as impulses patients take into f2f sessions | to code when the therapist intends to use TONI to enable the patient to gain new perspectives or impulses | *My experience with patients who do the modules is that they get impulses again that might otherwise go unnoticed, or they read more deeply into things on their own. So it really stimulates this... so it very much activates the patients' self-work. I find that very, very good. (T1)* |  |
|  | intervention characteristics | Therapist's knowledge about TONI platform (modules, functions) and assessment of the contents relevance for decisions regarding the participation inclusion | to code when therapist reports how much knowledge they have about the platform (e.g. not even looked at it or tested all contents themselves once) and whether TONI platform is relevant for their decision to invite patients | *Well, no, I wouldn't say that, because I think that I find suitable modules for every patient. So I don't think there are individual patients where I think it doesn't fit at all. So no, I would not say that I find an exclusion criterion due to the offer of the modules. (V7)* |  |
|  | psychoeducation | systematic, structured, and didactic transfer of knowledge about symptomatology and disorder and its treatment, integrating emotional and motivational aspects to enable patients to cope with the illness and to improve its treatment adherence and efficacy (Ekhtiari et al., 2017) | to code when the therapist intends to use TONI to provide psychoeducational content | *My main goal with TONI would be to provide the patients with a lot of information and a lot of possibilities to help themselves a bit, um... and the many contents, which are... within the therapy - that's not much, even if they come weekly, it's just 50 minutes a week - the many contents on what do the thoughts do, what do the emotions do, what is useful to know there in the sense of psychoeducation - so that's the main benefit, so to speak, that I expect (T7)* |  |
| **Temporal Aspects** | | Frequency of therapy, time restrictions within the study, time constraints of therapists |  |  |  |
|  | additional effort | Usage of TONI platform causes additional work for the therapist, e.g. through preparation and review | to code when therapist reports additional effort for themselves due to the application of TONI  not to code when additional effort for the patient is mentioned | *As of now, I would not include many patients at the same time, because it is enormously important for me to read what the patients write there. Because the modules, the building blocks that I release, I have to be very clear about "what is there again or what is being taught or what can we refer to?" And of course that takes time and I plan for that time. And that already, uh, of course makes a difference. (T7)* |  |
|  | moment of inclusion | The timing of inclusion in the study (after 1st session) | to code when therapist mentions that the timing of invitation (after the 1st session) and for registration in TONI platform influences the selection process | *And that's the problem, of course, that this is a study. For the TONI people it has to be like that, obviously, otherwise, it is somehow no longer manageable or measurable. For the practitioner, however, it would be much nicer to be able to do this at some point in the course of treatment. Not that the hurdle of taking these steps, of registering, for some patients, and the more heavily burdened they are, the higher the hurdle, that is already a goal. So if people manage to register somewhere and then re-register and complete the registration and fill out a questionnaire, that is already an enormous step for some patients. And for the study, it is somehow a prerequisite to participate. (V2)* |  |
|  | time saving | saving time by outsourcing certain contents, exercises etc. | to code when time savings in the therapeutic process are achieved with the help of TONI. For example, to be able to work on different contents in therapy in parallel with the help of TONI, or to intensify something that has been worked on in therapy  not to code when time savings for the patient (e.g. no travel time) are mentioned | *And I also see that it is possible through such things... Like I also had a case where a short-term therapy with only 12 sessions was sufficient. And I find that exciting. That you can save therapy sessions and achieve your goal more quickly, so that it somehow fits into these sometimes very tightly scheduled life plans. (T6)* |  |
| **Therapeutic Alliance** | | Importance of certain factors in the (just emerging) therapeutic relationship |  |  |  |
|  | stability of therapeutic relationship | Therapist's perception of the stability of the therapeutic relationship including doubts and uncertainty of the patient about the therapy | to code when the degree of stability of the therapeutic alliance is mentioned, also: to code when distrust in the therapeutic alliance is mentioned so sustainability is not established yet | *But I think with psy-, psychoform cases, with compulsive behavior, I don't think I would do it yet. The problem would actually be that I would be afraid that I would hurt the patients a bit, or that they would not feel taken seriously if I came to them with some (eh) video stories, yes (V4).* |  |
|  | submissiveness/favor | Therapist's impression that patients would participate to please the therapist rather than for themselves | To code if therapist mentions the impression that patient wants to please in the relationship | *think I would hesitate with someone who was very submissive in the contact, where I would have the feeling that they were not free to make their own decision, where I would have the strong feeling - especially now with the study participation... No, they would do it to do me a favour (T2)* |  |

**Appendix C**

**Results Coding System**

**Table 1**

*Number of Subcodes separated by Sample*

| **Code** | | **n total [1x/i]** | **n CBT [1x/i]** | **n PDT [1x/i]** |
| --- | --- | --- | --- | --- |
| **Therapist-related Factors** | |  |  |  |
| **Previous experience with digital interventions** | |  |  |  |
|  | none | 3/14 | 1/7 | 2/7 |
|  | little | 9/14 | 6/7 | 3/7 |
|  | routinely | 2/14 | 0/7 | 2/7 |
| **Evaluation of earlier experiences** | |  |  |  |
|  | low participation | 8/14 | 4/7 | 4/7 |
|  | useful | 7/14 | 4/7 | 3/7 |
| **Motivation to participate/use TONI** | |  |  |  |
|  | positive attitude towards TONI | 6/14 | 2/7 | 4/7 |
|  | supporting research | 6/14 | 2/7 | 4/7 |
|  | interest in expanding therapeutic possibilities | 14/14 | 7/7 | 7/7 |
| **Patients included in TONI** | |  |  |  |
|  | included | 10/14 | 6/7 | 4/7 |
|  | not included | 4/14 | 1/7 | 3/7 |
| **Patient-related factors** | |  |  |  |
| **Demographics** | |  |  |  |
|  | gender | 2/14 | 0/7 | 2/7 |
|  | age | 11/14 | 5/7 | 6/7 |
| **Living conditions of patients** | |  |  |  |
|  | technical equipment | 8/14 | 3/7 | 5/7 |
|  | safe workstation | 8/14 | 2/7 | 6/7 |
|  | time resources | 10/14 | 4/7 | 6/7 |
| **Symptoms** | |  |  |  |
|  | anxiety and depression | 6/14 | 3/7 | 3/7 |
|  | autism | 2/14 | 0/7 | 2/7 |
|  | dissociative or psychotic symptoms | 9/14 | 4/7 | 5/7 |
|  | level of personality functioning | 3/14 | 0/7 | 3/7 |
|  | magnitude of acute and general distress | 10/14 | 6/7 | 4/7 |
|  | media addiction | 2/14 | 0/7 | 2/7 |
|  | severe trauma | 6/14 | 2/7 | 4/7 |
| **Patients' preconditions** | |  |  |  |
|  | ability to reflect | 2/14 | 2/7 | 0/7 |
|  | cognitive abilities | 5/14 | 2/7 | 3/7 |
|  | fit for outpatient care | 11/14 | 6/7 | 5/7 |
|  | fluency in German (written and spoken) | 6/14 | 3/7 | 3/7 |
|  | Internet literacy | 14/14 | 7/7 | 7/7 |
|  | participation motivation | 12/14 | 6/7 | 6/7 |
|  | therapeutic experience | 2/14 | 0/7 | 2/7 |
| **Therapy and Study Conditions** | |  |  |  |
| **Role of intervention for PT** | |  |  |  |
|  | give impulses | 7/14 | 1/7 | 6/7 |
|  | intervention characteristics | 12/14 | 7/7 | 5/7 |
|  | psychoeducation | 5/14 | 2/7 | 3/7 |
| **Temporal aspects** | |  |  |  |
|  | additional effort | 5/14 | 3/7 | 2/7 |
|  | moment of inclusion | 10/14 | 5/7 | 5/7 |
|  | time saving | 8/14 | 5/7 | 3/7 |
| **Therapeutic alliance** | |  |  |  |
|  | stability of therapeutic alliance | 13/14 | 7/7 | 6/7 |
|  | Submissiveness / favor | 2/14 | 0/7 | 2/7 |

*Note.* Codes were only counted once per participant [1x/i].

**Table 2**

*Overview of Subcode and Valence Code Combinations Regarding Patient Inclusion*

| **Codes** | | **n total [1x/i]** | **prerequisite or exclusion (1x/i)** | **prerequisite (1x/i combination valence and criterion)** | **exclusion (1x/i combination valence and criterion)** | **indifferent (1x/i combination valence and criterion)** | **usage adaptation (1x/i combination valence and criterion)** | **Particularly suitable (1x/i combination valence and criterion)** |
| --- | --- | --- | --- | --- | --- | --- | --- | --- |
| **Patient-related factors** | |  |  | | | | | |
| **Demographics** | |  |  |  |  | CBT: 4/7 |  |  |
|  | age | 11/14 | CBT: 2/7 PDT: 1/7 | CBT: 1/7 (PDT: 2/7 due to study conditions >=18 years) | CBT: 1/7 PDT: 1/7 | CBT: 4/7 PDT: 1/7 |  | PDT: 3/7 |
|  | gender | 2/14 |  |  |  | PDT: 1/7 |  |  |
| **Living conditions of patients** | |  |  |  |  | CBT: 4/7 |  |  |
|  | technical equipment | 8/14 | CBT: 3/7  PDT: 4/7 | PDT: 3/7 | CBT: 3/7  PDT: 3/7 |  | PDT: 1/7 |  |
|  | safe workstation | 8/14 | CBT: 1/7  PDT: 4/7 | PDT: 2/7 | CBT: 1/7  PDT: 2/7 | CBT: 1/7  PDT: 2/7 | PDT: 1/7 |  |
|  | time resources | 10/14 | CBT: 2/7  PDT: 4/7 | CBT: 2/7  PDT: 1/7 | PDT: 3/7 |  | CBT: 1/7  PDT: 1/7 | CBT: 1/7  PDT: 1/7 |
| **Symptoms** | |  |  |  |  | CBT: 1/7 |  |  |
|  | anxiety & depression | 6/14 |  |  |  |  | CBT: 1/7  PDT: 2/7 | CBT: 2/7  PDT: 1/7 |
|  | autism | 2/14 | PDT: 1/7 |  | PDT: 1/7 |  |  | PDT: 1/7 |
|  | dissociative or psychotic symptoms | 9/14 | CBT: 4/7  PDT: 5/7 |  | CBT: 4/7  PDT: 5/7 |  | PDT: 1/7 |  |
|  | level of personality functioning | 3/14 | PDT: 1/7 |  | PDT: 1/7 | PDT: 2/7 | PDT: 1/7 | PDT: 1/7 |
|  | magnitude of acute and general strain | 10/14 | CBT: 5/7  PDT: 3/7 | CBT: 1/7 | CBT: 5/7  PDT: 3/7 | PDT: 1/7 | PDT: 2/7 | CBT: 2/7  PDT: 1/7 |
|  | media addiction | 2/14 | PDT: 1/7 |  | PDT: 1/7 |  | PDT: 1/7 |  |
|  | severe trauma | 6/14 | CBT: 2/7  PDT: 3/7 |  | CBT: 2/7  PDT: 3/7 |  | PDT: 2/7 |  |
| **Patients' preconditions** | |  |  | | | | | |
|  | ability to reflect | 2/14 | CBT: 2/7 | CBT: 2/7 |  |  | CBT: 1/7 |  |
|  | cognitive abilities | 5/14 | CBT: 2/7  PDT: 2/7 | CBT: 1/7  PDT: 1/7 | CBT: 1/7  PDT: 2/7 | PDT: 1/7 | CBT: 1/7 |  |
|  | fit for outpatient care | 11/14 | CBT: 5/7  PDT: 5/7 | CBT: 4/7  PDT: 5/7 | CBT: 2/7 |  | CBT: 1/7 |  |
|  | Fluency in German (written and spoken) | 6/14 | CBT: 3/7  PDT: 3/7 | CBT: 3/7  PDT: 3/7 | CBT: 1/7  PDT: 1/7 |  |  | PDT: 1/7 |
|  | Internet literacy | 14/14 | CBT: 6/7  PDT: 5/7 | CBT: 2/7  PDT: 3/7 | CBT: 4/7  PDT: 2/7 | CBT: 1/7  PDT: 2/7 | CBT: 1/7  PDT: 1/7 | CBT: 1/7  PDT: 2/7 |
|  | participation motivation | 12/14 | CBT: 3/7  PDT: 6/7 | CBT: 2/7  PDT: 3/7 | CBT: 3/7  PDT: 4/7 | PDT: 1/7 | CBT: 1/7 | CBT: 2/7 |
|  | therapeutic experience | 2/14 | PDT: 2/7 |  | PDT: 2/7 | PDT: 1/7 | PDT: 1/7 | PDT: 1/7 |
| **Therapeutic alliance** | |  |  | | | | | |
|  | stability of therapeutic alliance | 13/14 | CBT: 4/7  PDT: 4/7 | CBT: 2/7 | CBT: 2/7  PDT: 4/7 | CBT: 3/7  PDT: 4/7 | CBT: 1/7  PDT: 1/7 | PDT: 1/7 |
|  | submissiveness / favour | 2/14 | PDT: 1/7 |  | PDT: 1/7 |  | PDT: 1/7 |  |

*Note.* Code combinations were only counted once per participant [1x/i], multiple combinations of subcodes and valence codes are possible for one participant. When coding, not every subcode was assigned a valence code.
